# Crystallize It before It Diffuses: Kinetic Stabilization of Thin-Film Phosphorus-Rich Semiconductor CuP_2_

**DOI:** 10.1021/jacs.2c04868

**Published:** 2022-07-13

**Authors:** Andrea Crovetto, Danny Kojda, Feng Yi, Karen N. Heinselman, David A. LaVan, Klaus Habicht, Thomas Unold, Andriy Zakutayev

**Affiliations:** † Materials Science Center, National Renewable Energy Laboratory, Golden, Colorado 80401, United States; ‡ Department of Structure and Dynamics of Energy Materials, Helmholtz-Zentrum Berlin für Materialien und Energie GmbH, 14109 Berlin, Germany; § Department Dynamics and Transport in Quantum Materials, Helmholtz-Zentrum Berlin für Materialien und Energie GmbH, 14109 Berlin, Germany; ∥ Material Measurement Laboratory, National Institute of Standards and Technology, Gaithersburg, Maryland 20899, United States; ⊥ Institute of Physics and Astronomy, University of Potsdam, 14476 Potsdam, Germany

## Abstract

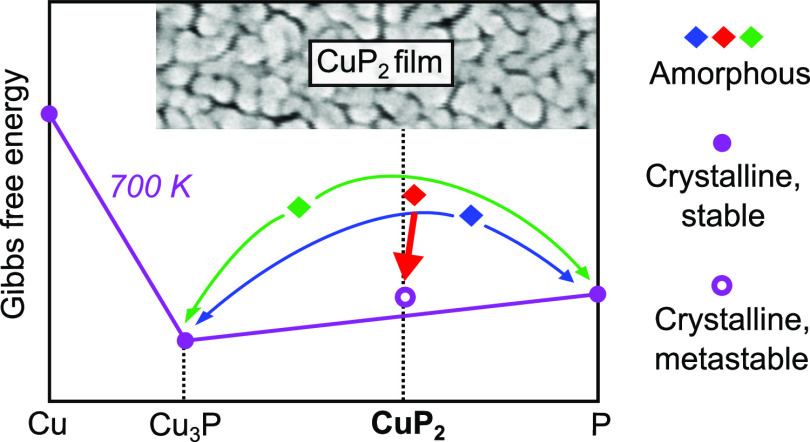

Numerous phosphorus-rich metal phosphides containing both P–P bonds and metal–P bonds are known from the solid-state chemistry literature. A method to grow these materials in thin-film form would be desirable, as thin films are required in many applications and they are an ideal platform for high-throughput studies. In addition, the high density and smooth surfaces achievable in thin films are a significant advantage for characterization of transport and optical properties. Despite these benefits, there is hardly any published work on even the simplest binary phosphorus-rich phosphide films. Here, we demonstrate growth of single-phase CuP_2_ films by a two-step process involving reactive sputtering of amorphous CuP_2+*x*_ and rapid annealing in an inert atmosphere. At the crystallization temperature, CuP_2_ is thermodynamically unstable with respect to Cu_3_P and P_4_. However, CuP_2_ can be stabilized if the amorphous precursors are mixed on the atomic scale and are sufficiently close to the desired composition (neither too P poor nor too P rich). Fast formation of polycrystalline CuP_2_, combined with a short annealing time, makes it possible to bypass the diffusion processes responsible for decomposition. We find that thin-film CuP_2_ is a 1.5 eV band gap semiconductor with interesting properties, such as a high optical absorption coefficient (above 10^5^ cm^–1^), low thermal conductivity (1.1 W/(K m)), and composition-insensitive electrical conductivity (around 1 S/cm). We anticipate that our processing route can be extended to other phosphorus-rich phosphides that are still awaiting thin-film synthesis and will lead to a more complete understanding of these materials and of their potential applications.

## Introduction

Phosphorus readily forms homoelement bonds in the solid state. Accordingly, over 100 phosphorus-rich binary metal phosphides containing both P–P bonds and metal–P bonds have been synthesized in bulk form.^1,2^ Often, these compounds have semiconducting properties and decompose into elemental phosphorus and a metal-rich phosphide (with only metal–P bonds) at high temperatures.^2^ Thin-film synthesis of P-rich materials would help determine their technological potential and their compatibility with established materials and processes. In addition, growing these materials in thin-film form would be desirable for high-throughput characterization of their properties as a function of composition and process conditions. However, reports of polycrystalline P-rich phosphides as thin films are very scarce and seem to be limited to basic characterization of ZnP_2_ and CdP_2_ deposited by evaporation of powders of the presynthesized compounds.^3,4^ Thin-film growth from elemental or gaseous sources would significantly simplify the synthesis process. However, the high P partial pressure required to stabilize these P-rich compounds poses additional challenges for thin-film growth with respect to bulk synthesis. The classic method of heating the elements in powder form in a sealed ampule cannot easily be extended to phosphorization of metal thin films. Because the volume of a thin film is very small, it is difficult to achieve a sufficiently high P partial pressure without excessive P recondensation on the film. On the other hand, open-system processes with fixed gas flow rates are more controllable, but the combination of a high P partial pressure, high temperature, and a continuously flowing P source requires careful safety measures.

Like many other P-rich phosphides, bulk synthesis of CuP_2_ as a single-crystal or powder is well established,^5−10^ but there are no reports of thin-film growth. CuP_2_ is a semiconductor that has been proposed as a solar absorber,^11^ thermoelectric material,^12,13^ electrocatalyst for hydrogen and oxygen evolution,^14^ and a component in composite anode materials for Li-^15−17^ and Na-based batteries.^18,19^ Although CuP_2_ has been incorporated in electrochemical devices, its optoelectronic and thermoelectric characterization is incomplete. For example, the optical absorption coefficient of CuP_2_ crystals has only been measured in the weak absorption region just above its 1.4–1.5 eV band gap,^8,10^ so it is impossible to evaluate its performance as a light absorber in the visible region. For thermoelectric applications, the properties needed to calculate the quality factor *zT* have only been measured separately on different CuP_2_ specimens in single-crystal or powder form. A potential method for growing phosphorus-rich phosphide thin films is reactive sputtering. We have recently shown the feasibility of this deposition technique for various metal-rich phosphide compounds.^20−22^

In this work, we present a relatively simple two-step process route to grow polycrystalline CuP_2_ thin films as semiconductors of potential technological interest. First, we deposit amorphous CuP_2+*x*_ by reactive sputtering in a PH_3_-containing atmosphere. The advantage of this process step is that sufficient P can be incorporated in the films at room temperature at a relatively low PH_3_ partial pressure (0.1 Pa). In the second step, we crystallize the CuP_2+*x*_ films by rapid thermal annealing (RTA) at atmospheric pressure under an inert gas flow. With this two-step process, high phosphorus partial pressures at high temperatures are avoided. We find that the crystallization step must be kinetically facilitated by employing amorphous precursors of sufficiently similar composition to the desired CuP_2_ stoichiometry. We investigate the optical properties of CuP_2_ over a broad spectral range and conduct comprehensive temperature-dependent thermoelectric characterization (including the *zT* value) up to room temperature. We find a remarkably high optical absorption coefficient (above 10^5^ cm^–1^ in the visible region), low thermal conductivity (1.1 W/(K m)), composition-insensitive electrical conductivity (1 S/cm), and a moderate native doping density (10^15^–10^17^ cm^–3^) potentially suitable for photovoltaic applications.

## Results and Discussion

### Structure and Bonding

**Figure 1:**
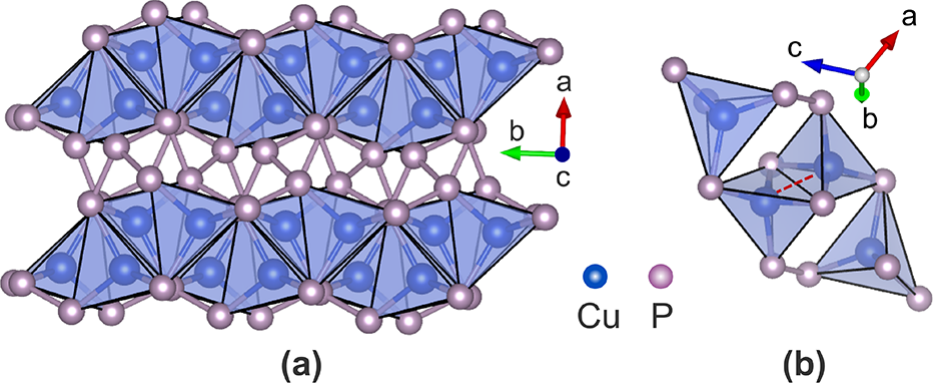
Monoclinic *P*2_1_/*c* structure of CuP_2_. (a) View emphasizing the sheets of CuP_4_ tetrahedra and of single-bonded P atoms. (b) View emphasizing the short Cu–Cu distance (dashed line) between two edge-sharing CuP_4_ tetrahedra.^23^

Bonding in CuP_2_ has some interesting features that are worth a brief analysis. Bulk CuP_2_ crystallizes in the monoclinic structure shown in Figure 1, with space group *P*2_1_/*c*.^7^ The structure consists of alternating sheets of CuP_4_ tetrahedra and of homoelement-bonded P atoms in planes parallel to (100) (Figure 1). Each CuP_4_ tetrahedron shares an edge and three corners with other analogous tetrahedra. The existence of anion–anion bonding is a key qualitative difference between P-rich compounds like CuP_2_ and most optoelectronic compounds such as III–V and II–VI semiconductors. The generalized 8 – *N* rule^24^ can then be used to interpret bonding. In this framework, one may assign the −1 oxidation state to one-half of the P atoms, since they are bonded to two other P atoms and three Cu atoms. The remaining P atoms have three P–P bonds and one Cu–P bond and are formally neutral, as the three homoelement bonds complete their octet. To achieve charge neutrality, Cu should then be in the +1 oxidation state. While explicit calculations^12^ indicate that only about 30% of this charge is actually transferred to P due to significant covalency, they also confirm that the charge is only accepted by the P atoms that are in the −1 oxidation state. Thus, CuP_2_ is a relatively rare example of a compound with mixed anion valence.

Another peculiar feature of the *P*2_1_/*c* structure of CuP_2_ is that pairs of Cu atoms are quite close to each other (2.48 Å).^7^ Comparing this distance to the bond length of metallic Cu (2.55 Å) and the metallic radius of single-bonded Cu (2.49 Å)^25^ suggests that some metallic Cu–Cu bonding is to be expected. This is confirmed by calculation of a nonzero electron localization function between the two Cu atoms and by experimental analysis of phonon modes in CuP_2_.^26^ These Cu–Cu dimers were recently shown to vibrate anharmonically as a rattling mode and strongly scatter acoustic phonons.^26^ This is the key feature enabling low lattice thermal conductivity in CuP_2_ in spite of its relatively high acoustic conductivity, thus making it interesting for thermoelectrics.

### Synthesizability

**Figure 2:**
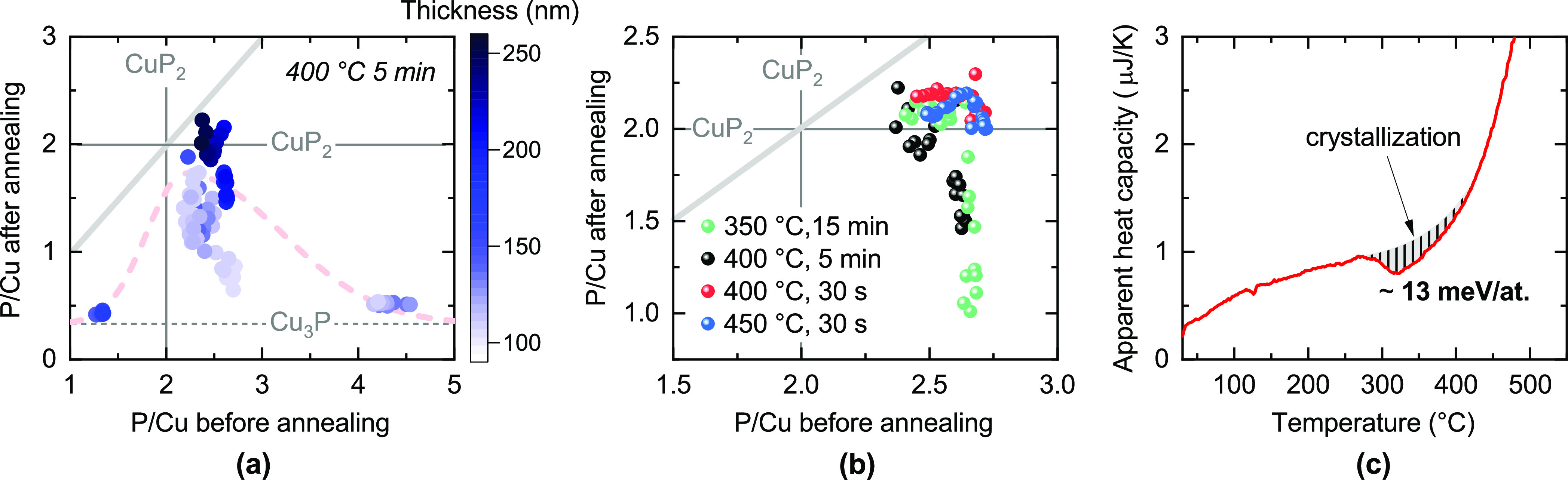
Effect of postannealing on amorphous CuP_2+*x*_ films. (a) Change in P/Cu ratio after annealing for films of different initial compositions and thicknesses under constant annealing conditions (400 °C for 5 min). Vertical and horizontal lines indicate the CuP_2_ and Cu_3_P stoichiometries. The gray diagonal line corresponds to P/Cu ratios that are not modified by annealing. The dashed line is a guide to the eye, indicating that the films with initial composition closest to CuP_2_ are the ones with least severe P losses. (b) Change in P/Cu ratio after annealing for films of CuP_2.4_–CuP_2.7_ initial composition and similar thicknesses under different annealing conditions. (c) Calorimetry experiment on an initially amorphous 90 nm thick CuP_2.5_ film deposited on a Si_3_N_4_ membrane. If we assume a baseline for the heat capacity as shown in the figure, the energy released in the 300–400 °C region (area under the baseline) is estimated as 13 meV/atom.

CuP_2+*x*_ thin films with a broad range of *x* (positive and negative) could be deposited by reactive sputtering in a PH_3_/Ar atmosphere at room temperature by using either a Cu target, a Cu_3_P target, or both at the same time (see the Experimental Details and the *x*-axis in Figure 2a). The main available parameters to tune *x* are the RF power on the targets and the PH_3_ partial pressure (see the Supporting Information). Decreasing the power led to higher P contents due to a more P-enriched target surface and/or to a lower flux of Cu at the substrate, promoting phosphorization at the substrate. Higher PH_3_ partial pressures can be achieved by increasing the total pressure or the PH_3_ concentration in Ar. Because the PH_3_ concentration was limited to below 5% in our setup, we had to employ a relatively high sputter pressure (2 Pa ≃ 15 mTorr) to obtain films of CuP_2_ stoichiometry. The wide tunability of the P content in Cu–P films was also observed in our recently reported amorphous B–P films by reactive sputtering.^21^ This compositional flexibility is likely related to the ability of P to form homoelement bonds in the films and segregate as an elemental impurity. Hence, we assume that the excess P in CuP_2+*x*_ films with *x* > 0 is mainly bonded to other P atoms.

As-deposited CuP_2+*x*_ films did not exhibit any X-ray diffraction (XRD) peaks (Figure S2), so we crystallized them in an RTA furnace at atmospheric pressure under a N_2_ flow. Loss of phosphorus at moderate temperatures is a well-known phenomenon in many P-rich phosphides.^2^ We also observed P losses in all our postannealed CuP_2_ films (Figure 2a,b). However, the dependence of these P losses on the initial composition of the as-deposited films is not trivial.

In Figure 2a, we compare the P/Cu ratio before and after annealing at 400 °C for 5 min for various initial compositions between CuP_1.3_ and CuP_4.5_. The P/Cu ratio is measured by X-ray fluorescence (XRF) so it represents an average through the depth of the film. Several interesting trends can be identified. First, thicker films generally experience milder P losses because P located deeper in the film requires a longer time to diffuse out. Second, sufficiently thick films with initial composition in the CuP_2.2_–CuP_2.7_ range can be “locked” into the desired CuP_2_ stoichiometry by annealing (Figure 2b). Third, films with severe P losses tend to approach the Cu_3_P composition after annealing. Cu_3_P is the most commonly reported binary stoichiometry in the Cu–P system.^5,6,27,28^

Last, and most surprisingly, we find that highly P-rich initial compositions do not help achieve a higher P content in the postannealed films. In fact, the opposite is true. When the initial composition is in the (P-rich) CuP_4.1_–CuP_4.5_ range, the postannealed composition is around CuP_0.5_ (Figure 2a). When the initial composition is much poorer in P (CuP_1.3_–CuP_1.4_ range), the postannealed composition is similar, around CuP_0.4_ (Figure 2a). On the other hand, when the initial composition is in an intermediate CuP_2.2_–CuP_2.7_ range closer to the desired CuP_2_ stoichiometry, P losses upon annealing are much slower in films of comparable thickness.

Using these atomically dispersed precursors with moderate P excess with respect to the target CuP_2_ stoichiometry, the necessary species for forming crystalline CuP_2_ are readily available within a subnanometer distance of their ideal crystallographic site. This enables fast crystallization of monoclinic CuP_2_. On the other hand, solid-state diffusion processes responsible for P losses have longer characteristic lengths, on the order of the film thickness (in our case, hundreds of nanometers). Thus, there is an optimal annealing time that is sufficient for CuP_2_ to crystallize but insufficient for substantial P losses to occur. Presumably, the lower total energy achieved by crystallizing the originally amorphous CuP_2_ film (Figure 2c) also helps delay P evaporation.

**Figure 3:**
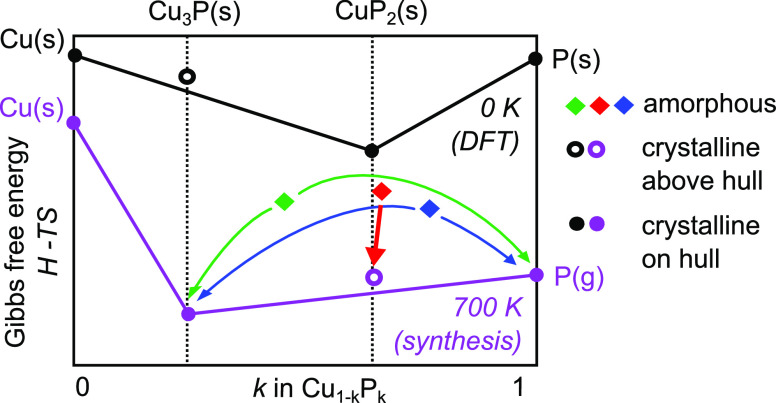
Qualitative convex hull of the Cu–P system at two different temperatures. Among competing phases, only Cu_3_P and the elements are considered. At 0 K, the Gibbs free energy only consists of enthalpy *H*, so the convex hull is drawn following DFT enthalpy calculations as available on the Materials Project database.^27^ CuP_2_ is found to be a stable phase (on the convex hull), and Cu_3_P is slightly metastable (above the convex hull). At 700 K, our experiments indicate that CuP_2_ is destabilized. Part of the reason may be a large entropic term *TS* for elemental P, which is in gaseous form at this temperature. When higher energy amorphous precursors (diamond data points) are rapidly heated to 700 K, decomposition into Cu_3_P and gaseous P is thermodynamically favored. However, CuP_2_ formation is kinetically facilitated when the initial composition of the precursors is sufficiently close to the CuP_2_ stoichiometry (red diamond).

Conversely, precursor films that are too P-rich require solid-state diffusion to form a crystalline CuP_2_ phase because Cu atoms are too far apart in the initial amorphous phase. This Cu diffusion process now competes with the unwanted P diffusion leading to P evaporation. As a result, P losses are much faster. These findings are summarized in the qualitative diagram shown in Figure 3.

**Figure 4:**
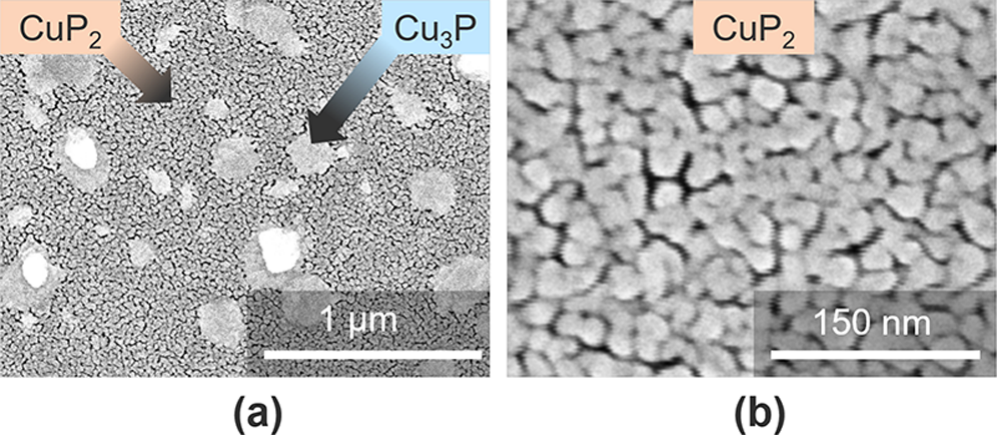
Morphology of a postannealed film with overall CuP_1.3_ composition. (a) Low-magnification SEM image showing a dual-phase morphology with a CuP_2_ matrix and Cu_3_P islands. (b) High-magnification SEM image showing porosity in the CuP_2_ matrix. Phase identification was performed on the basis of spatially resolved EDX spectra (Figure S1).

To visualize the P loss process, we image a film with final composition CuP_1.3_ by scanning electron microscopy (SEM, Figure 4). Two phases can be clearly distinguished on the micrometer scale: a porous polycrystalline matrix with grain size around 30 nm and islands of more compact morphology. The intensity ratio between the Cu and the P peaks in energy-dispersive X-ray spectroscopy (EDX) increases by a factor ∼5.5 when moving from the matrix to the islands (Figure S1). Thus, we conclude that the matrix consists of CuP_2_ and the islands consist of Cu_3_P. The mechanism of conversion from CuP_2_ to Cu_3_P appears to be diffusion of Cu in the plane of the substrate, contributing to the enlargement of seed Cu_3_P islands. At the same time, P gradually evaporates elsewhere. Because the most stable gaseous form of phosphorus^29^ at our annealing temperatures is P_4_ and no intermediate solid phases between CuP_2_ and Cu_3_P are observed, the CuP_2_ decomposition reaction can be written as 12CuP_2_(s) → 4Cu_3_P(s) + 5P_4_(g).

Figure 2b shows the effect of annealing temperature and time on the final composition. As expected, increasing the annealing time at fixed temperature results in more severe P losses (compare the data from 30 s versus 5 min annealing time at 400 °C). In general, longer annealing times can be tolerated at lower annealing temperatures. For example, annealing at 350 °C for 15 min yields about as many P-poor samples as the case of annealing at 400 °C for 5 min. Note that the spread of final P/Cu ratios sometimes obtained for films of otherwise similar initial P/Cu ratios, and the thicknesses is mainly caused by the different positions of the samples inside the furnace. The samples located further downstream with respect to the gas flow tend to lose less P, possibly because they are exposed to a finite P partial pressure due to P evaporating from the samples further upstream.

We observe a decrease in the apparent heat capacity of an as-deposited CuP_2.5_ film at around 300 °C by nanocalorimetry (Figure 2c). This indicates an exothermic signal, which may be related to the transition from the amorphous to the (more stable) polycrystalline state. The heat capacity baseline needed to calculate the heat of crystallization is not straightforward to define. If we assume the baseline shown in Figure 2c, we estimate the crystallization energy of CuP_2_ as 13 meV/atom. Although the uncertainty on this value is substantial, some qualitative conclusions can still be drawn. The calculated formation enthalpy of CuP_2_ from the elements in their standard state is 112 meV/atom.^27^ Because this value is much larger than the estimated crystallization energy, most of the formation energy has probably already been released during formation of the amorphous compound. The thermal energy of a solid at 400 °C is approximately 3*kT* = 174 meV/atom by using the Dulong–Petit law. Thus, the extra stabilization achieved by crystallizing CuP_2_ is only a small fraction of the thermal energy available at that temperature.

It is also interesting to consider typical values for the calculated energy difference between the most stable amorphous configuration and most stable crystalline polymorph for a given material. This quantity has been calculated in a previous study for 41 material systems (mainly oxides) at 0 K.^30^ The energy difference varies between ∼50 and ∼500 meV/atom depending on the material. The significantly lower crystallization energy measured in CuP_2_ could indicate that the entropic contribution to the total energy is substantially higher in the amorphous state than in the crystalline state at finite temperatures. Higher entropy is indeed expected in the amorphous state due to higher disorder, and it would contribute to reducing the energy difference between the amorphous and crystalline state of CuP_2_ at ∼700 K with respect to 0 K. Although this explanation is plausible, it is also possible that CuP_2_ and other non-oxide compounds simply exhibit different energetic trends than the computationally investigated selection of compounds. Computational analysis of the energetics of a more diverse range of amorphous material systems would certainly be useful.

### Stability

Previous work on CuP_2_ single crystals does not comment on their stability under ambient conditions. On the basis of simple observations on our thin-film samples, we suggest that the air stability of CuP_2_ should be further investigated. A change in color is consistently observed in as-deposited CuP_2+*x*_ after few hours of exposure to ambient air, signaling a reaction that is not limited to a surface layer of a few nanometers thickness. For this reason, the films characterized in this work were annealed immediately after deposition. After annealing, the bulk properties of the films appear to be stable for a longer time (at least a few days) under ambient conditions, as judged by their visual appearance and electrical conductivity. The higher reactivity of amorphous CuP_2+*x*_ may be due to the extra P present before annealing and to the higher energy associated with the amorphous state (Figure 2c). Both the amorphous and the polycrystalline films appear to be stable in a N_2_ atmosphere.

After either type of film has been exposed to air for a sufficiently long time, the reaction front has reached the back surface of the film, as evident by visual inspection through the glass substrate. The exact details of the CuP_2+*x*_–air reaction are currently unknown. However, XRF measurements reveal a large decrease in P/Cu ratio after prolonged exposure to air, indicating that the reaction involves P losses. We suspect that the high sputter pressure (2 Pa) necessary to obtain a P/Cu ratio above 2 in our growth setup may explain why the reaction of CuP_2_ films with air is not limited to a surface layer. Films sputtered at high pressure are generally more porous and more air sensitive due to their higher surface area.^31^ Thus, we cannot conclude that CuP_2_ films are intrinsically unstable in air. The stability of CuP_2_ films sputtered at lower pressures or deposited by other techniques should be investigated to clarify this issue.

### Structural and Vibrational Properties

**Figure 5:**
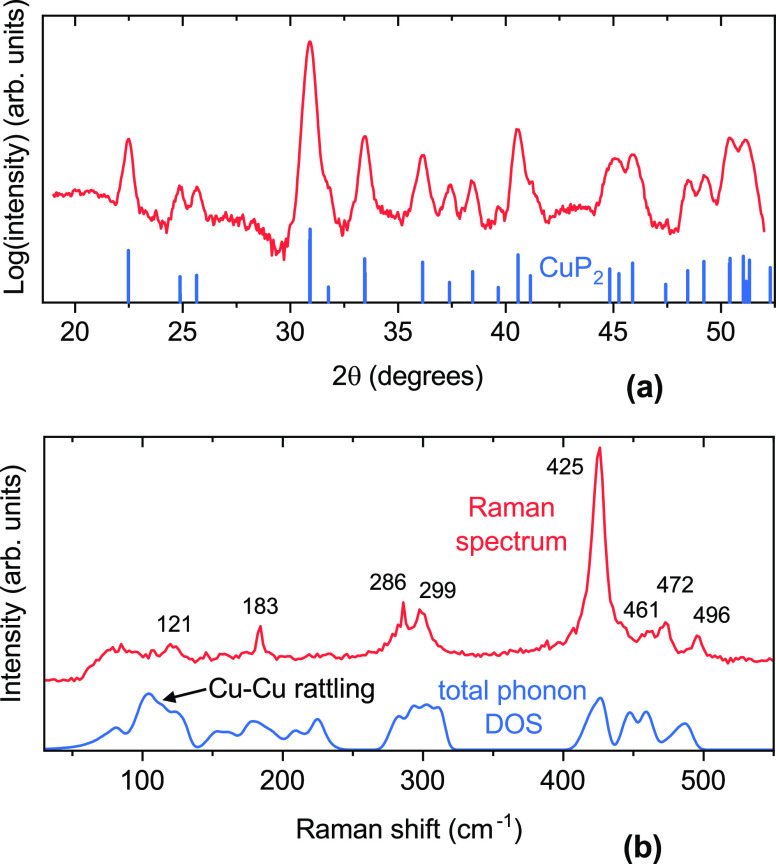
Structural and vibrational characterization of a film with CuP_2_ stoichiometry after postannealing at 450 °C for 30 s. (a) Experimental XRD pattern together with the reflections expected for randomly oriented CuP_2_ in the monoclinic *P*2_1_/*c* structure.^7^ XRD patterns under other annealing conditions are shown in Figure S2. (b) Experimental Raman spectrum with labels for the identified peak positions. The total phonon density of states of CuP_2_ in the *P*2_1_/*c* structure, as calculated in the Materials Project database,^27^ is also shown. The Cu–Cu rattling mode (∼100 cm^–1^) believed to limit the thermal conductivity of CuP_2_^26^ is indicated.

In agreement with nanocalorimetry results, the originally amorphous CuP_2+*x*_ films only begin to show crystalline XRD peaks above 300 °C annealing temperature (Figure S2). Beyond this lower limit, it is possible to obtain polycrystalline CuP_2+*x*_ films in the *P*2_1_/*c* structure under various annealing conditions. As long as the final composition is close to the nominal CuP_2_ stoichiometry, XRD patterns of films processed under different annealing conditions are rather similar (Figure S2). As an example, the XRD pattern of a CuP_2_ film annealed at 450 °C for 30 s (Figure 5a) contains all the peaks expected for the *P*2_1_/*c* structure, without major preferential orientation effects and without clear peaks from secondary phases above the noise level. The XRD peak positions closely match the positions of the reference bulk CuP_2_ sample,^7^ indicating that structural parameters (including the short Cu–Cu distance) are about the same in thin-film and bulk CuP_2_. XRD peaks from Cu_3_P in the hexagonal *P*6_3_*cm* structure are observed in CuP_2+*x*_ films when *x* < 0 (Figure S2). However, the threshold value of *x* at which Cu_3_P peaks begin to appear strongly varies with annealing conditions. When *x* > 0 (P-rich films), no XRD peaks associated with secondary phases like Cu_2_P_7_ or elemental phosphorus are observed up to the most P-rich composition reached in this study (CuP_2.2_).

**Figure 6:**
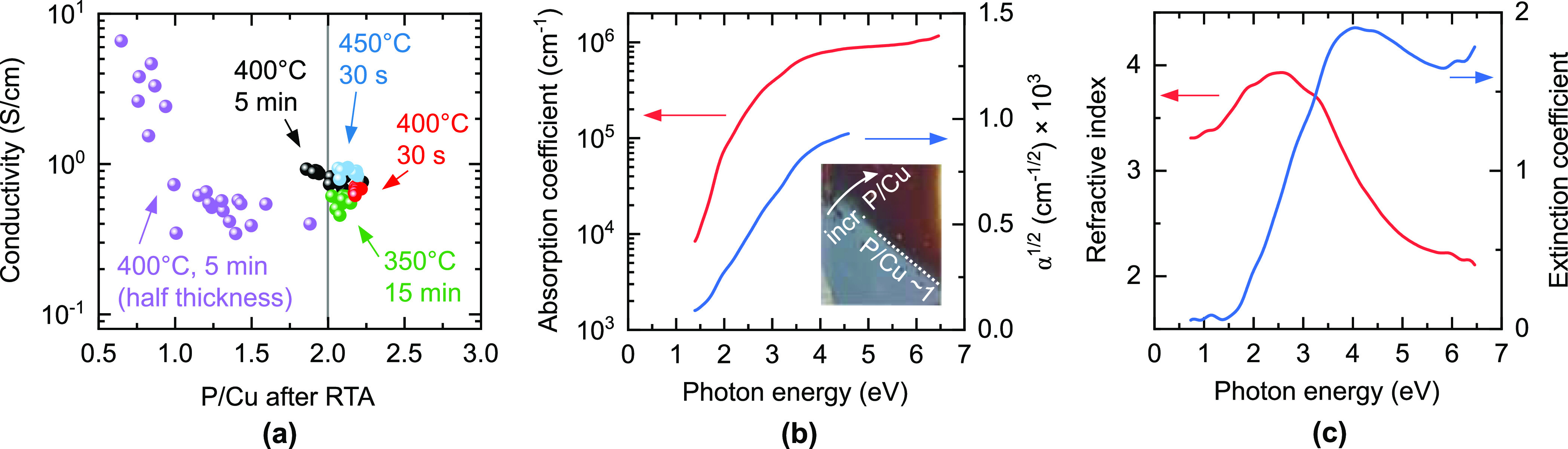
Room-temperature electrical and optical properties of postannealed CuP_2+*x*_ films. (a) Electrical conductivity as a function of composition (measured after annealing) and annealing conditions. Films are of comparable thickness except for the P-poor purple data points, which have about half the thickness as the other ones. A zoomed-in view around the CuP_2_ stoichiometry is available in Figure S4. (b) Absorption coefficient α of a postannealed CuP_2.0_ film together with a α^1/2^ plot versus photon energy. Inset: photograph of a film with increasing P/Cu ratio from bottom left to top right. When the P/Cu ratio decreases below roughly 1, the appearance of the film changes from dark red (characteristic of semiconducting CuP_2_) to gray (characteristic of metallic Cu_3_P). (c) Refractive index and extinction coefficient of the same CuP_2.0_ film shown in (b).

It might be tempting to conclude that single-phase CuP_2+*x*_ can be grown over a wide *x* range, in which defect formation is favored over secondary phase precipitation. However, secondary phases in amorphous form (not detected by XRD) are likely to be present in our samples for the following reasons. First, amorphous secondary phases were identified in a previous study on the phase equilibrium between Cu_3_P and CuP_2_ in powder form.^32^ This observation rendered XRD-determined phase boundaries incorrect. Second, our short annealing times may not be sufficient to crystallize phases with a significantly different composition than the original precursors. In fact, Cu_3_P can only form if CuP_2_ loses more than 80% of its original P in some regions of the film. If the annealing process is stopped before these losses can take place, and if there are no stable phases between CuP_2_ and Cu_3_P, the likely result is formation of amorphous phases with intermediate composition. Third, the electrical conductivity of our films is roughly constant in the CuP_1.0_–CuP_2.2_ composition range (Figure 6a). It is improbable that the high defect densities required to accommodate this nonstoichiometry do not have any effect on the electrical properties. Thus, electrically inactive secondary phases (such as the disconnected Cu_3_P islands shown in Figure 4a) are very likely to coexist with point defects in highly nonstoichiometric CuP_2_.

The Raman spectrum of the same sample used for XRD characterization is plotted in Figure 5b. The phonon density of states (DOS) of CuP_2_, as calculated by density functional perturbation theory in good agreement with recent experiments,^26^ is also shown for comparison.^27,33,34^ Because Raman spectra of bulk CuP_2_ are not available in the literature, we briefly discuss some qualitative aspects here. Raman features originating from the phonon bands centered around 300 and 450 cm^–1^ can clearly be seen in the experimental spectrum. In particular, the most intense Raman peak at 425 cm^–1^ probably arises from one of the lowest-energy phonon branches within the highest-energy band in the calculated DOS. All modes in this band essentially involve vibrations of P atoms with nearly static Cu atoms. The lower the phonon energy, the larger the contribution from Cu vibrations, as expected from the larger mass of Cu.

Because the film is polycrystalline, there are selection rules for Raman-active phonon modes and the Raman spectrum will not directly reflect the phonon DOS. Specifically, all atoms in CuP_2_ are at 4e Wyckoff positions of the *P*2_1_/*c* space group, so only the A_g_ and B_g_ modes are Raman-active according to the character tables.^35^ With a 12-atom unit cell, 18 Raman-active modes are predicted in total.^35^ Eight peaks can be identified the experimental spectrum (Figure 5b). The Cu–Cu rattling mode identified by Qi et al. as an important scatterer of heat-transporting phonons^26^ is either symmetry-forbidden or too low in intensity to be distinguished by Raman spectroscopy.

### Electrical and Optical Properties

The room-temperature electrical conductivity of postannealed polycrystalline films in the CuP_2.0_–CuP_2.2_ composition range is between 0.5 and 1.0 S/cm at room temperature, without a clear dependence on the P/Cu ratio (Figure 6a). The conductivity slightly increases with increasing annealing temperature, regardless of annealing time (Figure S4). Previously reported conductivities of CuP_2_ single crystals range from 0.01 to 30 S/cm, presumably due to differences in the crystal quality.^6,8−10^ Films with severe P losses have significantly higher conductivities (Figure 6a), probably due to percolation paths between highly conductive Cu_3_P phases.^6^ The Seebeck coefficient measured on a freshly annealed CuP_2.0_ film is +390(10) μV/K (Figure S3), indicating native p-type doping. All previously reported CuP_2_ single crystals were also p-type with higher Seebeck coefficients in the 690–820 μV/K range. The work function, measured with a Kelvin probe in air on a freshly annealed CuP_2.0_ film, is 5.0(1) eV.

CuP_2_ is a relatively strong absorber of light. Its absorption coefficient α reaches 10^5^ cm^–1^ at a photon energy *hν* = *E*_g_ + 0.6 eV above its band gap *E*_g_ = 1.5(1) eV (Figure 6b). This compares favorably even with the most efficient direct gap photovoltaic absorbers such as GaAs, CdTe, and CH_3_NH_3_PbI_3_ (MAPI).^36^ In fact, the absorption coefficient is as high as in some exciton-enhanced photoabsorbers such as BiI_3_ and Cu_2_BaSnS_4_,^37,38^ indicating that CuP_2_ may deserve more detailed optoelectronic characterization.

We find that α^1/2^ is linear in photon energy over a 2 eV spectral range above the band gap (Figure 6b), indicating that α ∝ (*hν* – *E*_g_)^2^. Both the estimated band gap and the spectral dependence of the absorption coefficient are in agreement with previous work on CuP_2_ single crystals.^8−10^ Because the α ∝ (*hν* – *E*_g_)^2^ behavior is often associated with an indirect gap in conventional semiconductors,^39^ an indirect gap was previously assumed for these CuP_2_ crystals.^8,10^

However, there are at least two other factors to consider. (1) The absorption strength of CuP_2_ is high even for a direct gap material, so indirect transitions are unlikely to be responsible for it. (2) According to the calculated band structure of CuP_2_,^27^ the fundamental gap should be direct and located between the Γ and the Y point of the Brillouin zone. Two indirect gaps with slightly higher energies exist due to additional valence band pockets at the X point and between the Y and H points.^27^ Even though we observe an α ∝ (*hν* – *E*_g_)^2^ behavior, care should be taken when employing the absorption characteristics typical of group IV and III–V semiconductors to interpret the nature of the optical transitions of other semiconductors with substantially different band structures. A clear difference between CuP_2_ and conventional semiconductors is that the former has many valence and conduction band pockets at different points of the Brillouin zone. Hence, many different optical transitions can contribute to the overall absorption coefficient.

The refractive index of CuP_2_ is 3.3–3.4 in the transparent region (Figure 6c). Extrapolation of the real part of the dielectric function to zero photon energy (Figure S5) yields a high-frequency permittivity ε_*∞*_ = 10.5 ± 1.0. Interestingly, there seems to be a critical P/Cu ratio close to 1, where the electrical and optical properties shift from being “CuP_2_-like” (semiconducting and IR transparent) to being “Cu_3_P-like” (metallic and opaque). This transition is manifested by an abrupt change in conductivity (Figure 6a) and visual appearance (inset of Figure 6b).

### Thermoelectric Characterization

We conducted DC and double AC Hall effect measurements as well as temperature-dependent thermoelectric characterization of three films. They have the following compositions: Cu_2.50_P (labeled “Cu_3–*z*_P”), Cu_1.61_P, and CuP_1.35_ (labeled “CuP_2–*y*_”). We use these labels to emphasize similarity to Cu_3_P and CuP_2_ as discussed in the previous section. This set of films was deposited on Si_3_N_4_ membranes as part of a microchip-based thin-film transport characterization platform.^40^ Differences between this set of films and the films deposited on glass characterized in the rest of the article are listed in the Supporting Information. Because these films have intermediate compositions between CuP_2_ and Cu_3_P, their properties may be influenced by inhomogeneity, as exemplified by the dual-phase morphology shown in Figure 4. Nevertheless, important qualitative trends in the transport properties of these films as a function of composition can still be discerned.

**Figure 7:**
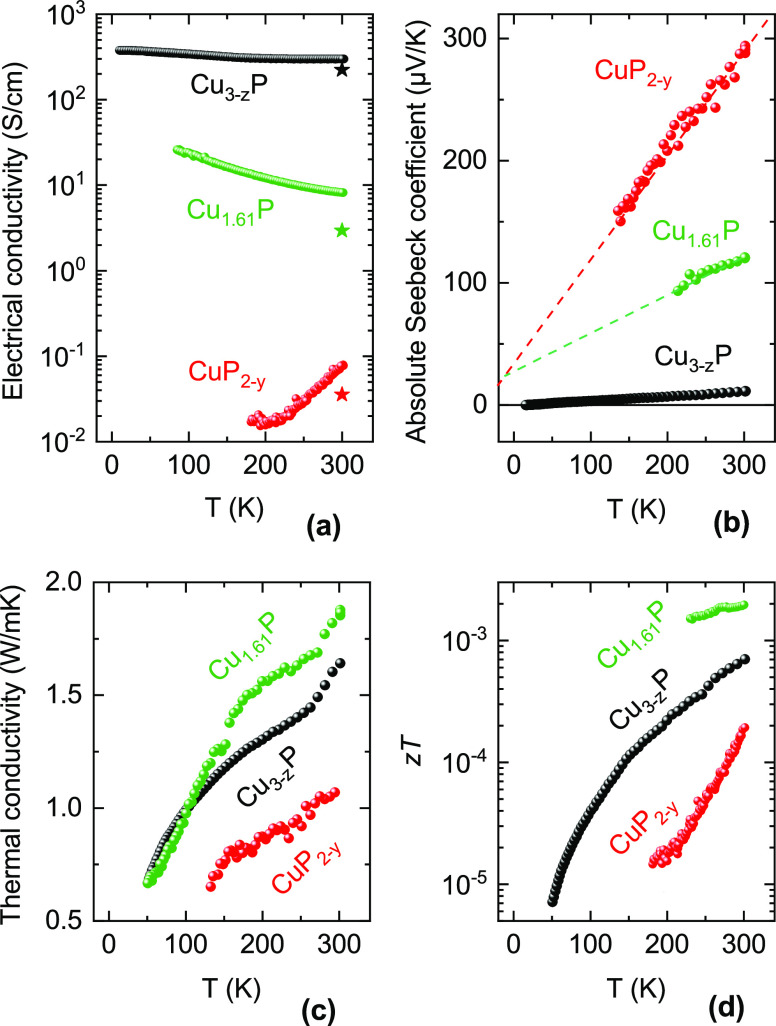
Thermoelectric properties of three postannealed Cu–P films as a function of temperature *T*. The compositions after annealing are indicated. (a) Electrical conductivity σ, which was also remeasured at room temperature one month after the temperature-dependent measurement (star markers). (b) Absolute Seebeck coefficients *S* ≡ *S*_Cu–P_, with linear trends indicated. (c) Thermal conductivity κ. (d) Thermoelectric figure of merit *zT* = σ*S*^2^*T*/κ.

**Figure 8:**
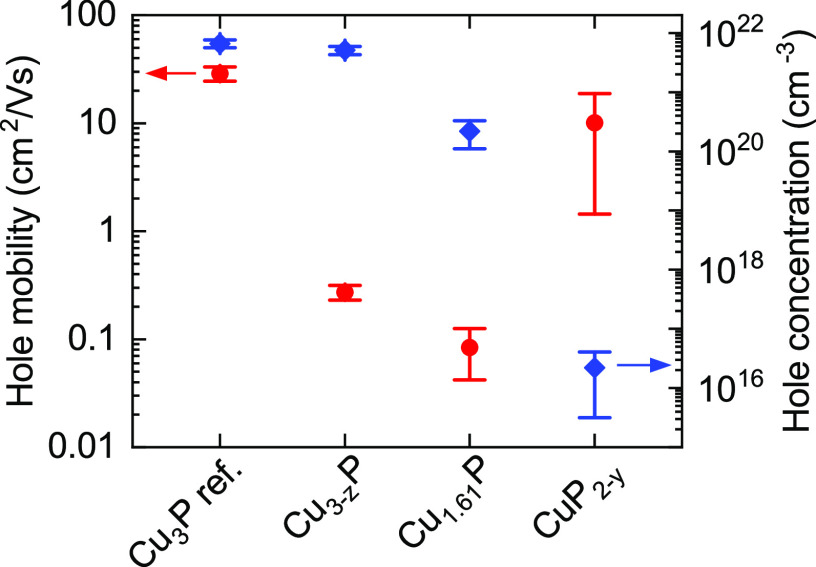
Hole mobility and concentration by double AC Hall effect measurements on the same Cu–P films shown in Figure 7. The film labeled “Cu_3_P ref.” is a continuous polycrystalline Cu_3_P film deposited by reactive sputtering at 360 °C and used as a reference.

The temperature dependence of the electrical conductivity (Figure 7a) suggests that CuP_2–*y*_ is a nondegenerately doped semiconductor and that the two other films are either metallic or degenerately doped semiconductors. Hall effect measurements at room temperature confirm this interpretation (Figure 8), with high carrier concentrations measured in Cu_3–*z*_P and Cu_1.61_P (above 10^20^ cm^–3^) and a moderate carrier concentration measured in CuP_2–*y*_ (10^15^–10^17^ cm^–3^). All films have a positive Hall voltage confirming their p-type conductivity. Note that the conductivity of Cu_1.61_P and CuP_2–*y*_ after one month of storage is appreciably lower (Figure 7a), highlighting possible stability issues as discussed in the previous sections.

Because of the inverse relationship between carrier concentration and thermovoltage,^41^ the Seebeck coefficient is highest in CuP_2–*y*_ and lowest in Cu_3–*z*_P (Figure 7b). Interestingly, the Seebeck coefficient increases linearly with temperature in all three films (Figure 7b). This behavior is often a sign of a temperature-independent carrier concentration,^41^ a typical feature of materials with nonzero density of states at the Fermi level (i.e., metals and degenerate semiconductors such as Cu_3–*z*_P and Cu_1.61_P). However, a linear increase of the Seebeck coefficient with temperature is not readily explained for a more weakly doped semiconductor like CuP_2–*y*_. In an ideal scenario, we would expect the carrier concentration to increase with temperature due to increasing defect ionization and the Seebeck coefficient to decrease accordingly. The reason for this discrepancy is unclear. One could invoke the role of film inhomogeneity due to the presence of Cu_3_P secondary phases (Figure 4) or assume that the increase of electrical conductivity with temperature (Figure 7a) is due to mobility changes rather than to the hole concentration changes. Yet, a simultaneous increase in hole concentration and Seebeck coefficient with temperature was reported for CuP_2_ single crystals,^8^ where inhomogeneity effects can be excluded. Multiband transport could also cause an unusual temperature behavior due to increasing contributions from the additional valence band pockets of CuP_2_ with increasing temperature. However, application of the Boltzmann transport equation^42^ on the calculated CuP_2_ band structure^27^ reveals that a significant decrease in the Seebeck coefficient is expected in the 200–300 K range assuming a concurrent increase in hole concentration by 1 order of magnitude (Figure S6).

As another hypothesis, one could assume that CuP_2–*y*_ is highly compensated by donor defects at low temperatures, but its p-type character becomes more dominant at higher temperatures due to activation of a deeper acceptor. If this hypothesis is correct, one would expect both the electrical conductivity and the Seebeck coefficient to increase with temperature as we experimentally observe—the former due to an increase in the concentration of ionized acceptors and the latter due to a decreasing contribution from the (negative) n-type Seebeck coefficient.^41^ Previous work also suggested the possibility of charge compensation in CuP_2_ single crystals based on the temperature dependence of their carrier mobility.^10^ The position of the acceptor level in our CuP_2–*y*_ film can be estimated as 121(3) meV above the valence band from an Arrhenius plot of the electrical conductivity in the 230–300 K temperature range (Figure S7).

The room-temperature thermal conductivity of CuP_2–*y*_ is 1.1 W/(K m) (Figure 7c). This value is lower than in CuP_2_ single crystals (3.6–4.7 W/(K m) depending on lattice direction)^26^ as may be expected for a polycrystalline sample. Our measured conductivity is, however, in excellent agreement with the calculated 1.12 W/(K m) amorphous limit for bulk CuP_2_.^12^ The increasing thermal conductivity with increasing temperature is unlike the ∝1/*T* behavior typical of crystalline semiconductors in this temperature range. Instead, it is often observed in amorphous or highly disordered materials, consistent with the observation that our measured conductivity is very close to the amorphous limit. On the basis of these results, we assume that the phonon mean free path in the CuP_2–*y*_ film is low due to disorder^43^ and/or phonon boundary scattering.^44^ The latter is likely enhanced by the small grains, low thickness, and porous morphology of the film.^43−45^ The electronic contribution to the thermal conductivity is negligible due to the low hole concentration of CuP_2–*y*_ (Figure 8). The thermal conductivities of Cu_3–*z*_P and Cu_1.61_P are only slightly higher and their temperature dependences are similar to the case of CuP_2–*y*_. Thus, we conclude that the thermal conductivity is phonon-mediated and strongly limited by film morphology in all three films. In fact, scattering of charge carriers (holes) is also morphology-limited. The hole mobility of the present Cu_3–*z*_P film (0.27 cm^2^/(V s)) is 2 orders of magnitude lower than in a continuous Cu_3_P film on glass with about the same carrier concentration (28.8 cm^2^/(V s), see Figure 8).

CuP_2_ has recently been proposed as a potential thermoelectric material.^12,13^ Our measurements on a CuP_2–*y*_ film confirm that the lattice contribution to its thermal conductivity is indeed sufficiently low for thermoelectric applications. However, the thermoelectric figure of merit *zT* at room temperature is still low for all investigated compositions (Figure 7d) due to low power factors (Figure S8). In the vicinity of the Cu_3_P stoichiometry, the main issue is a low Seebeck coefficient. In the vicinity of the CuP_2_ stoichiometry, the main issue is low electrical conductivity. Even taking the more favorable properties of our CuP_2_ films on glass (Table 1) or of previously reported CuP_2_ single crystals,^8^ the *zT* value at room temperature would only be 0.004 and 0.05, respectively. It might be possible to optimize the hole concentration of CuP_2_ by extrinsic doping to obtain higher *zT* values. Nevertheless, phosphorus losses at moderate temperatures and potential stability issues under ambient conditions are likely to limit its practical applicability in thermoelectric devices. Similar issues might exist in other phosphorus-rich phosphides.

**Table 1:** List of Electrical, Optical, and Thermal Properties Measured in This Study on Postannealed CuP_2+*x*_ Films at Room Temperature^a^

electrical conductivity	0.5–1.0	S/cm
Seebeck coefficient	+390 ± 10	μV/K
thermal conductivity	1.1 ± 0.1	W/(K m)
band gap	1.5 ± 0.1	eV
work function	5.0 ± 0.1	eV
dielectric constant (ε_*∞*_)	10.5 ± 1.0	

a The film composition is CuP_2.0_ for all properties except for the thermal conductivity (CuP_1.35_).

## Conclusion

We deposited amorphous CuP_2+*x*_ thin films with a wide range of *x* (positive and negative) by reactive sputtering in a PH_3_/Ar atmosphere. By annealing these films above their crystallization temperature in an inert atmosphere, we observed that the CuP_2_ phase was thermodynamically unstable with respect to the Cu_3_P phase. However, it was possible to kinetically stabilize polycrystalline CuP_2_ by satisfying all the following conditions: (1) amorphous precursors mixed on the atomic level (rather than a heterogeneous mixture of amorphous components) to ensure the correct local bonding environment; (2) initial composition sufficiently close to the ideal P/Cu ratio of 2, also to ensure the correct local bonding environment; (3) annealing temperature just high enough to allow for solid-state diffusion; (4) annealing time just long enough for crystallization to be completed, but not long enough for a large fraction of P to diffuse to the surface.

Remarkably, amorphous films that were either too P-poor or too P-rich quickly decomposed into Cu_3_P and gaseous phosphorus upon heating. This “compositional lock-in” behavior highlights the importance of pre-existing short-range order for kinetic stabilization of materials under conditions where decomposition and crystallization are in competition with each other.

Polycrystalline CuP_2+*x*_ films are semiconductors with native p-type conductivity. Their electrical properties are rather insensitive to elemental composition in the vicinity of the stoichiometric point and only moderately affected by the annealing conditions. The thermal conductivity of a P-poor CuP_2_ film is 1.1 W/(K m) at room temperature, confirming its potential applicability as a thermoelectric material. However, the hole conductivity of CuP_2_ is too low to achieve a high power factor (and therefore a high *zT* value) without extrinsic doping. Furthermore, decomposition of CuP_2_ into Cu_3_P and gaseous phosphorus at around 400 °C hinders high-temperature applications. Although stability issues are not mentioned in the CuP_2_ single-crystal literature, our polycrystalline CuP_2+*x*_ films were only stable in ambient conditions for a few days. It is currently not clear if this issue is related to the porous morphology of our films or if it is an intrinsic behavior of CuP_2_.

Finally, CuP_2_ is a stronger light absorber than many established photovoltaic materials, with absorption coefficient rapidly rising to 10^5^ cm^–1^ above its 1.5 eV band gap. Combined with a native doping density in the optimal range for a photovoltaic absorber in a pn junction solar cell (10^15^–10^17^ cm^–3^), we conclude that CuP_2_ may deserve more detailed optoelectronic characterization.

## Experimental Details

### Film Growth

Amorphous CuP_2+*x*_ thin films were deposited on Corning Eagle XG borosilicate glass by reactive radio frequency (RF) sputtering over a 10 × 5 cm^2^ area. A Cu target and a Cu_3_P target were cosputtered at 2 Pa total pressure in a 5% PH_3_/Ar atmosphere without intentional heating and without substrate rotation. The targets were oriented so that one short side of the substrate would mainly be coated by the Cu target and the other short side by the Cu_3_P target.

Immediately after deposition, CuP_2+*x*_ films were cut into smaller pieces and annealed in a lamp-based rapid thermal annealing (RTA) furnace in a N_2_ atmosphere. Because of the sputtering target geometry and differences in their applied power, small gradients in P/Cu ratio and film thickness were obtained across the substrate. These gradients enabled us to characterize several data points (“samples”) for each annealing run, each with a distinct composition and thickness. More details on film deposition and annealing are available in the Supporting Information.

### Film Characterization

All measurements except for nanocalorimetry and thermoelectric/Hall effect characterization were performed within 24 h after annealing to avoid sample degradation. The combinatorial characterization data arising from compositional gradients in the films were managed with the COMBIgor tool^46^ and the Research Data Infrastructure^47^ and integrated into the High-Throughput Experimental Materials Database.^48^

Elemental composition and film thickness were determined by X-ray fluorescence (XRF) calibrated by Rutherford backscattering spectrometry (RBS, composition) and spectroscopic ellipsometry (thickness). X-ray diffraction (XRD) measurements were conducted by using Cu Kα radiation, a 2D detector, and a fixed incidence angle of 10°. Raman spectra were measured with 532 nm excitation wavelength and 4 W/mm^2^ power density. Scanning electron microscopy (SEM) images were taken at 5 kV beam voltage.

Sheet resistance was measured with a collinear four-point probe directly contacting the film. The Seebeck coefficient of a CuP_2_ film on glass was measured in a custom-built setup by using In contacts. The work function was measured with a Kelvin probe calibrated with a standard Au sample. The absorption coefficient and optical functions were extracted by spectroscopic ellipsometry. Because of higher porosity in the upper part of the film, we modeled the system as a glass substrate of known optical functions, a CuP_2_ layer with a linearly increasing fraction of air from bottom to top,^49^ and a roughness layer treated with Bruggeman effective medium theory.

For nanocalorimetry and thermoelectric/Hall effect characterization, CuP_2+*x*_ films were deposited on previously described microfabricated chips designed for calorimetry^50^ and in-plane thermoelectric characterization^40^ of thin-film samples. In both types of chips, CuP_2+*x*_ was deposited on a free-standing Si_3_N_4_ membrane. Because of the fragility of the membrane, thinner CuP_2+*x*_ films (90–120 nm) were employed for these studies.

Nanocalorimetry experiments were conducted in a N_2_ atmosphere on an as-deposited amorphous film with initial CuP_2.5_ composition, with an average heating rate of roughly 5000 °C/s. Temperature-dependent thermoelectric characterization (electrical and thermal conductivity and Seebeck coefficient) was performed in a vacuum on three films with different compositions after annealing. The electrical conductivity was measured by using the van der Pauw (vdP) method.^51^ The Seebeck coefficient was measured with respect to platinum metals lines by using an internal four-probe platinum thermometer.^40^ The thermal conductivity was derived from the current–voltage characteristics of membrane heaters/thermometers in the self-heating regime.^40,52^ The hole concentration and mobility were measured on the same samples by double AC Hall.^53^ More details on all measurements are available in the Supporting Information.

## Supporting Information Available

EDX analysis of a multiphase film, XRD patterns as a function of composition and annealing conditions, electrical conductivity close to the ideal CuP_2_ stoichiometry, complex dielectric function spectra, RBS spectra used for calibration of elemental composition, simulation of Seebeck coefficient of CuP_2_, additional thermoelectric characterization data (PDF)
