# Supplementary material for: Crystallize It before It Diffuses: Kinetic Stabilization of Thin-Film Phosphorus-Rich Semiconductor CuP2
Source: J Am Chem Soc. 2022 Jul 13;144(29):13334–43. doi: 10.1021/jacs.2c04868 (PMC9335872; doi:10.1021/jacs.2c04868)
Supplement: Supplementary file 1 — ja2c04868_si_001.pdf [file ja2c04868_si_001.pdf]

SUPPORTING INFORMATION

Crystallize it before it diffuses: Kinetic stabilization of thin-film phosphorus-rich semiconductor CuP_2

Andrea Crovetto,[*,†,‡] Danny Kojda,[¶] Feng Yi,[§] Karen N. Heinselman,[†] David A. LaVan,[§] Klaus Habicht,[¶,‖] Thomas Unold,[‡] and Andriy Zakutayev[*,†]

†*Materials Science Center, National Renewable Energy Laboratory, Golden, Colorado 80401, United States*

‡*Department of Structure and Dynamics of Energy Materials, Helmholtz-Zentrum Berlin für Materialien und Energie GmbH, 14109 Berlin, Germany*

¶*Department Dynamics and Transport in Quantum Materials, Helmholtz-Zentrum Berlin für Materialien und Energie GmbH, 14109 Berlin, Germany*

§*National Institute of Standards and Technology, Material Measurement Laboratory, Gaithersburg, Maryland 20899, United States*

‖*Institute of Physics and Astronomy, University of Potsdam, 14476 Potsdam, Germany*

E-mail: Email:ancro@dtu.dk; Email:andriy.zakutayev@nrel.gov

Extended experimental details

Film growth

Amorphous CuP_{2+x} thin films were deposited on Corning Eagle XG borosilicate glass by reactive radio-frequency (RF) sputtering over a $10 \times 5\,\mathrm{cm}^2$ area in a PVD Products sputter system with base pressure in the 10^{-5} Pa range. A Cu target and a Cu_3P target (both 2″ diameter, 0.25″ thickness, 99.99% purity) were co-sputtered at 2 Pa total pressure in a PH_3/Ar atmosphere without intentional heating and without substrate rotation. To obtain a $CuP_{2.5}$ composition, we employed 5% PH_3 in Ar, a total flow rate of 0.028 L/min, and RF sputter powers of 15 W (Cu_3P) and 10 W (Cu). The target-substrate distance was 16 cm and the deposition rate was about $0.2\,\text{Å}\,\mathrm{s}^{-1}$. To obtain a lower P content, we decreased the PH_3 concentration at constant total pressure. To obtain a higher P content, we decreased the RF powers on both targets by the same proportion. The targets were oriented so that one short side of the substrate would mainly be coated by the Cu target and the other short side by the Cu_3P target. The P/Cu ratio of the film varied by about 10% across the long direction (10 cm long), confirming that the surfaces of both targets are in a similar phosphorus-enriched state, giving comparable deposition rates. Depositing CuP_2 at lower sputter pressures would require higher PH_3 concentrations which were not available in our setup. At 2 Pa sputter pressure, it was not possible to crystallize CuP_2 directly during sputter deposition by increasing the substrate temperature, due to severe P re-evaporation from the film. Substantially higher sputter pressures might prevent re-evaporation, but at the likely expense of deposition rate and film quality.

Due to the PH_3 gas used as a P source, it is possible that some H is incorporated in the as-deposited CuP_{2+x} films. Secondary ion mass spectrometry (SIMS) was used to measure the H concentration in amorphous Zn_3P_2 films in an earlier study.[1] Zn_3P_2 was deposited by reactive sputtering of a Zn target in a PH_3-containing atmosphere. The H concentration was 4 at.%. Due to the similarity with our process, similar concentrations of H may be present

in our amorphous as-deposited CuP_{2+x} films.

Immediately after deposition, CuP_{2+x} films were cut into smaller pieces and annealed in a lamp-based rapid thermal annealing (RTA) furnace (MILA-3000, ADVANCE RIKO, Inc.). The samples were placed on a Si susceptor in a quartz tube, the furnace was closed, purged with N_2 for 5 min, and the desired annealing steps were executed under an N_2 flow at 1 bar. For all processes discussed in the articles, the temperature was held at 150 °C for 1 min and then ramped up to the desired annealing temperature at 3 °C/s. The temperature was controlled through a feedback loop by monitoring the temperature of the Si susceptor. After annealing, the furnace was actively cooled with water and the samples were taken out at room temperature.

Characterization of films deposited on glass

All measurements except for nanocalorimetry and thermoelectric/Hall effect characterization were performed within 24 h after annealing to avoid sample degradation. Elemental composition and film thickness were determined by x-ray fluorescence (XRF) in a Bruker Tornado M4 instrument at 2×10^3 Pa pressure using a Rh source. XRF spectra were fitted with the Bruker XMethod analysis program. The composition was calibrated by Rutherford backscattering spectroscopy (RBS) measurements of separate CuP_{2+x} films of different thicknesses and compositions deposited on Si (Fig. S9). The thickness was calibrated by spectroscopic ellipsometry measurements of the same films (see below). RBS was performed using a model 3S-MR10 RBS system from National Electrostatics Corporation in a 168° backscattering configuration, using a 2 MeV He^+ beam. Samples were measured until the total integrated charge delivered to the sample was 80 μC, and signals were added together when multiple measurements were taken at a single point. Film composition from RBS was determined by fitting using the RUMP analysis software.[2]

X-ray diffraction (XRD) measurements were conducted with a Bruker D8 diffractometer using Cu K_α radiation, a 2D detector, and a fixed incidence angle of 10°. The diffraction

intensity at each 2θ angle was integrated over the χ range measured by the 2D detector. Raman spectra were measured with a Renishaw inVia Raman microscope under laser light excitation at 532 nm wavelength and $4\,W/mm^2$ power density. Scanning electron microscopy (SEM) images were taken with a Hitachi S-3400N instrument with a field emission gun and 5 kV beam voltage.

Sheet resistance was measured with a collinear four-point probe directly contacting the film, and electrical conductivity was derived from it using the XRF-determined thickness. The Seebeck coefficient of a CuP_2 film on glass was measured in a custom-built setup using In contacts and four temperature differences in the vicinity of room temperature. The work function was measured with a SKP SPV LE 450 Kelvin probe (KP Technology) calibrated with a standard Au sample.

Absorption coefficient and optical functions were extracted by spectroscopic ellipsometry using a J.A. Woollam M-2000 ellipsometer and three incidence angles. Due to higher porosity in the upper part of the film, we modeled the system as a glass substrate of known optical functions, a CuP_2 layer with a linearly increasing fraction of air from bottom to top,[3] and a roughness layer treated with Bruggeman effective medium theory. The optical functions of CuP_2 were represented by a Kramers-Kronig-consistent b-spline function with 0.1 nodes/eV. Ellipsometry spectra were fitted with the CompleteEase software (J.A. Woollam).

Nanocalorimetry and thermoelectric characterization of films deposited on Si_3N_4 membranes

For nanocalorimetry and thermoelectric/Hall effect characterization, CuP_{2+x} films were deposited on previously described microfabricated chips designed for calorimetry[4] and in-plane thermoelectric characterization[5] of thin-film samples. In both types of chips, CuP_{2+x} was deposited on a free-standing Si_3N_4 membrane. Due to the fragility of the membrane, thinner CuP_{2+x} films (90 nm–120 nm) were employed for these studies. Since these samples had to be shipped for measurements, they were packed in vacuum after their last processing step and

unpacked just before the measurement. Both calorimetry and thermoelectric measurements took place about a month after the samples had been packed.

Nanocalorimetry experiments were conducted in a N_2 atmosphere on an as-deposited amorphous film with initial $CuP_{2.5}$ composition. The film was heated to about 510 °C for a duration of 100 ms and the average heating rate is roughly 5000 °C/s. The heat released in the 300 °C–400 °C range was estimated as the area under the assumed heat capacity baseline divided by the volume of the film and the atomic density of CuP_2 in the $P2_1/c$ structure (0.062 atoms/Å^3).

Temperature-dependent thermoelectric characterization (electrical and thermal conductivity and Seebeck coefficient) was performed in vacuum on three films annealed under different conditions (composition before annealing: $CuP_{2.5}$). We found that phosphorus losses of these particular films during annealing were much faster than in the films deposited on glass. Two possible reasons are the lower thickness and better thermal contact with the Si susceptor during annealing. A $Cu_{2.50}P$ film (labeled "$Cu_{3-z}P$") was obtained by annealing at 400 °C for 30 s. A $Cu_{1.61}P$ film was obtained by annealing at 350 °C for 5 min. A $CuP_{1.35}$ film (labeled "CuP_{2-y}") was obtained by annealing at 350 °C for 30 s. Before thermoelectric characterization, the chips were glued to ceramic 24 pin dual in-line (DIL) chip packages using silver epoxy and wire bonded with aluminum wires. The packages were characterized in a tailored closed cycle refrigerator (Janis), providing vacuum down to $p = 7 \times 10^{-7}$ mbar, coaxial wiring to the packages, triple temperature shielding and a temperature range from 10 K–500 K. The setup and analysis routines were verified by reference measurements on 100 nm gold films.

The electrical conductivity of the Cu-P films was measured by current-voltage (I-V) lines[5] using the van der Pauw (vdP) method[6] and excitation currents of 100 µA ($Cu_{3-z}P$) and 1 µA ($Cu_{1.61}P$ and CuP_{2-y}). The I-V characteristics show linear behavior (see Fig. S10(a-c)) for the shown data range in Fig. 7(a) of the main article. Some of the low-temperature data was discarded due to a noisy vdP correction factor in $Cu_{1.61}P$ and high contact resistances

($> 1\times10^8\,\Omega$) in CuP_{2-y}, resulting in non-linear I-V characteristics.

For temperature-dependent Seebeck coefficient measurements, the Seebeck voltage $V_S(I_{\mathrm{heat}},T)$ and the resistance of the nearby four-probe platinum thermometer $R_{\mathrm{Pt}}(I_{\mathrm{heat}},T)$ were measured as function of different heating currents I_{heat} at bath temperature T.[5] The chip's individual thermometer calibration is given by $R_{\mathrm{Pt}}(I_{\mathrm{heat}}=0,T)$. Using this calibration, V_S (low potential on the cold side) can be expressed as function of the temperature difference ΔT_{S}. For all samples V_S was proportional to I^2_{heat} and ΔT_{S}, as expected and shown in Fig. S10(d-f). The relative Seebeck coefficient with respect to the platinum metals lines was found as $S_{\mathrm{Cu-P,Pt}} = S_{\mathrm{Cu-P}} - S_{\mathrm{Pt}} = -\partial V_S/\partial\Delta T_{\mathrm{S}}$. For calculation of the absolute Seebeck coefficient $S_{\mathrm{Cu-P}}$, reference data of Kockert *et al.* was used (sample PT3).[7] Some of the low-temperature data in Fig. 7(b) of the main article was discarded due to increasing contact resistance that hindered accurate voltage measurements.

For determination of the thermal conductivity, the I-V characteristics of both membrane heaters were measured with high resolution and with I_{sh} in the self-heating regime.[5] Here, the membrane heaters act as thermometers at the same time. Using the local derivative, the resistance R_{sh} can be expressed as function of the applied power P_{sh}. $R_{\mathrm{sh}}(P_{\mathrm{sh}})$ shows a linear relation as expected. The intercept of the linear regression of $R_{\mathrm{sh}}(P_{\mathrm{sh}})$ yield the individual thermometer calibration for both membranes. Using this calibration and considering that $\partial V/\partial I = R(P_{\mathrm{sh}}=0) + 3\Delta R_{\mathrm{sh}}$ the resistance change $\Delta R_{\mathrm{sh}}(P_{\mathrm{sh}})$ can be converted to $\Delta T_{\mathrm{sh}}(P_{\mathrm{sh}})$ for both membranes. Linear regression yield the thermal resistance $\partial P_{\mathrm{sh}}/\partial\Delta T_{\mathrm{sh}}$ for the large and the small membrane (Fig. S10(g-i)). Application of the math given in Völklein *et al.*[8] and measurements of the empty membrane yields the thermal conductivity of the film.

After thermoelectric characterization, the DIL packages were connected to the room temperature sample holder of a 8404 Hall measurement system (LakeShore). Using two MFLI lock-in amplifiers (Zurich Instruments AG) and a CS580 voltage driven current Source (Stanford Research Systems) the double AC Hall (DAC) method[9] was applied to significantly increase the signal to noise ratio. Our setup is verified by DC reference measurements on

InAs. Here, the frequency of the magnetic field $f_B = 0.8\,\text{Hz}$ and the frequency of the current excitation was set to $f_I = 77\,\text{Hz}$. For different excitation currents I_{ex} and different magnetic field B up to $0.3\,\text{T}$ the Hall voltage $V_H = V(f_I + F_B) + V(f_I - F_B)$ was measured. The linear relationship of $V_H(I_{\text{ex}} \cdot B)$ proves the Hall effect as origin and allows the determination of the carrier density. The error bars in Fig. 8 of the main article are derived from the standard error of the linear fits of V_H versus $(I_{\text{ex}} \cdot B)$ plots for each sample.

Supplementary figures

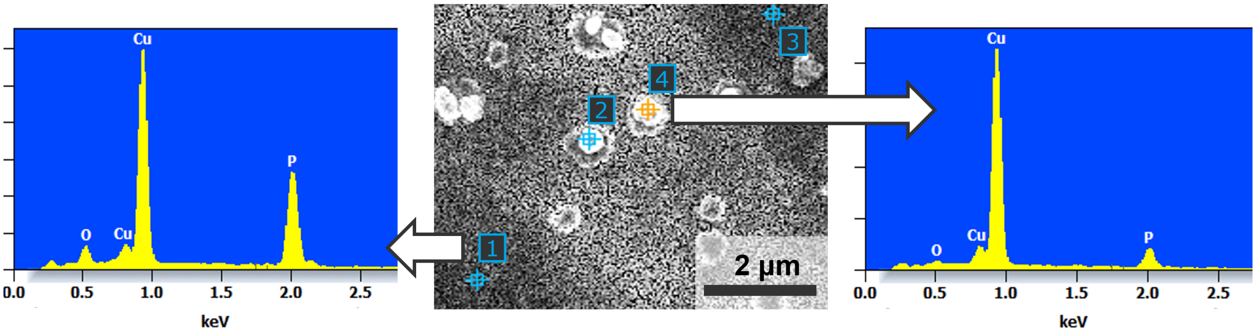

Figure S1: EDX point spectra on a post-annealed film with overall $CuP_{1.3}$ composition. The intensity ratio between the Cu L line and the P K line increases by a factor $\sim$ 5.5 when moving from the darker matrix to the brighter islands. Thus, we conclude that the matrix consists of CuP_2 and the islands consist of Cu_3P.

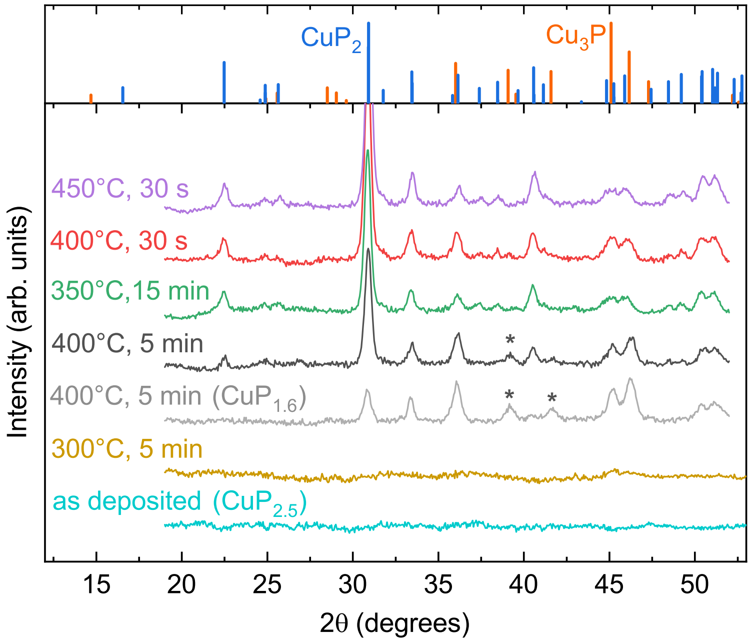

Figure S2: XRD patterns of CuP_{2+x} films annealed under different conditions. All films have $CuP_{2.0}$ composition after annealing, unless labeled otherwise. Reference reflections for CuP_2 in the $P2_1/c$ structure[10] and for Cu_3P in the $P6_3cm$ structure[11] are shown. The asterisks indicate Bragg reflections attributed to Cu_3P.

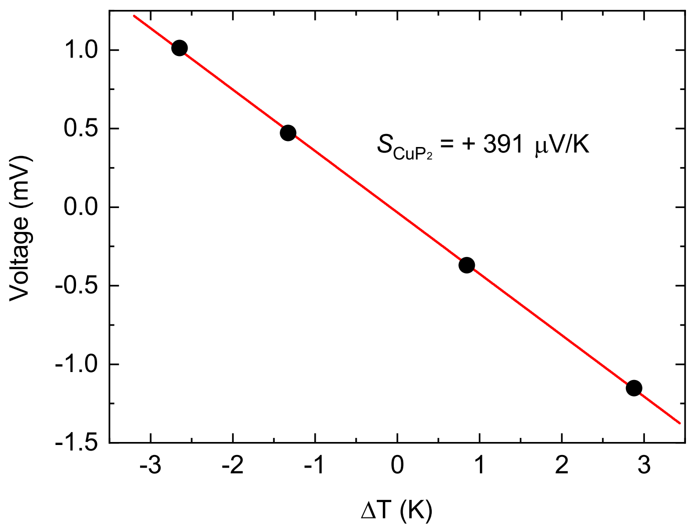

Figure S3: Thermovoltage measurements on a $CuP_{2.0}$ film on glass after annealing. The absolute Seebeck coefficient ($S_{\mathrm{CuP_2}}$) of $CuP_{2.0}$ is extracted as the slope of the fitted line with reverse sign, plus the known Seebeck coefficient of the In contacts used for the measurement, following the convention $S_{\mathrm{CuP_2,In}} = S_{\mathrm{CuP_2}} - S_{\mathrm{In}} = -\partial V_S/\partial \Delta T_S$.

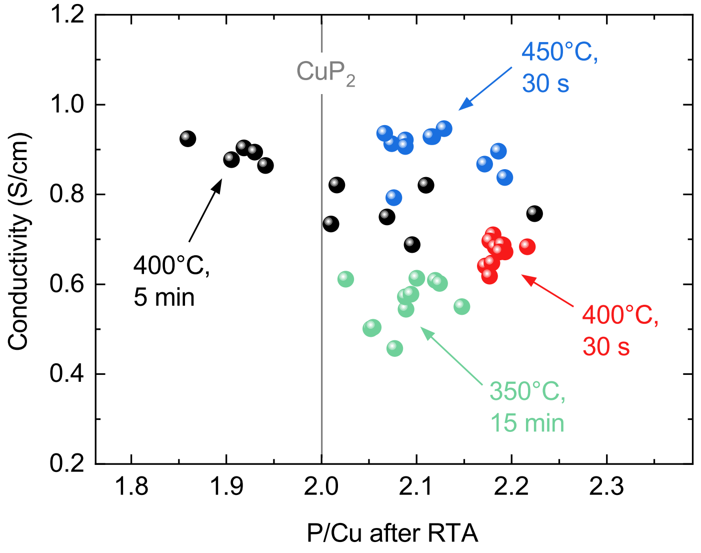

Figure S4: Electrical conductivity of post-annealed CuP_{2+x} films as a function of annealing conditions and final composition. The data is the same as in Fig. 6(a) of the main article, but here the focus is on the data points close to the P/Cu = 2 composition.

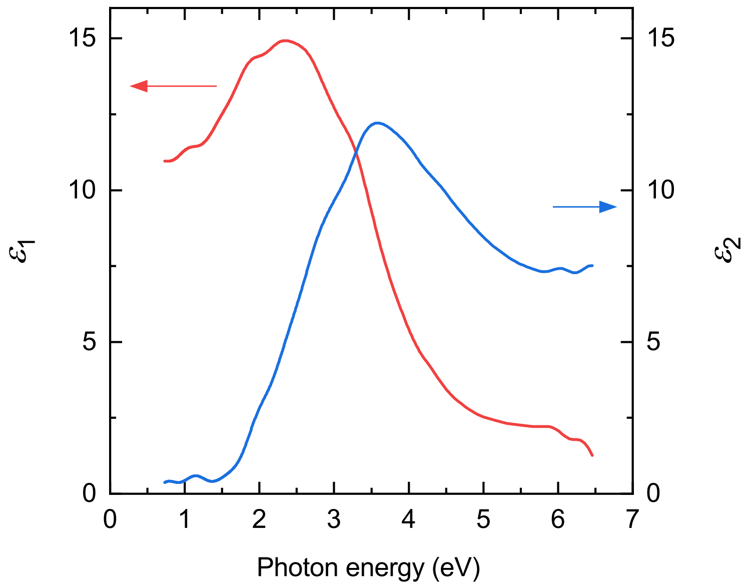

Figure S5: Real (ε_1) and imaginary part (ε_2) of the dielectric function of a $CuP_{2.0}$ film on glass after annealing. The spectra are derived from the ellipsometry-determined refractive index and extinction coefficient shown in Fig. 6(c) of the main article.

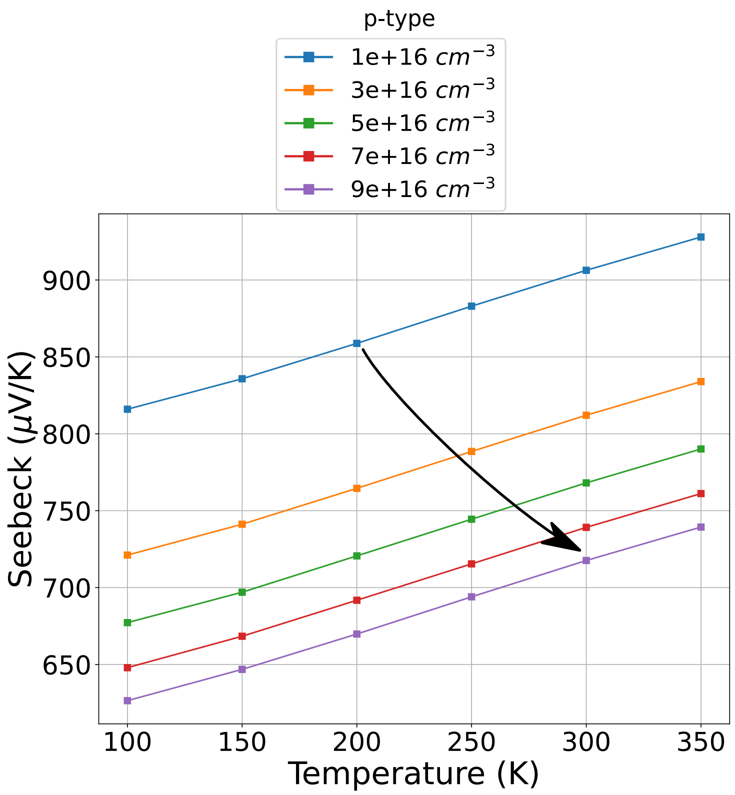

Figure S6: Calculated Seebeck coefficient of CuP_2 as a function of temperature. The Seebeck coefficient is determined by semiclassical Boltzmann transport theory using the BoltzTraP2 package[12] on the CuP_2 band structure. We employed the band structure calculated by density functional theory under the generalized gradient approximation (GGA) and available on the Materials Project database (mp-927).[13] The p-type Seebeck coefficient is calculated at different hole concentrations, indicated by colors. A hole relaxation time of 10 fs is assumed. We further assume that the increase in conductivity of the experimental CuP_{2-y} sample with temperature (Fig. 7(a), main article) is due to increasing hole concentration, which is in the $10^{16}\,\mathrm{cm}^{-3}$ range at room temperature (Fig. 8, main article). Under these assumptions, the arrow from $1 \times 10^{16}\,\mathrm{cm}^{-3}$ at 200 K to $9 \times 10^{16}\,\mathrm{cm}^{-3}$ at 300 K roughly indicates the expected temperature dependence of the Seebeck coefficient for a p-type CuP_2 crystal. The transport calculations include the possible influence of other valence band maxima energetically close to the absolute valence band maximum along the Γ–Y line.

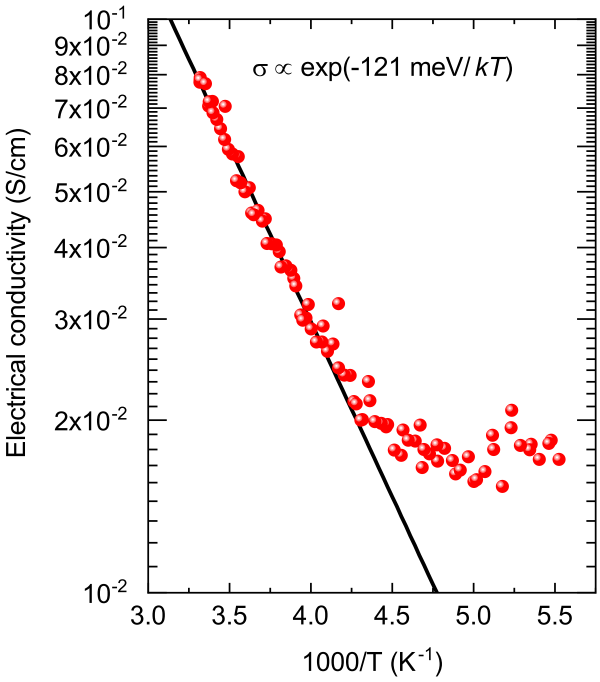

Figure S7: Arrhenius plot of the electrical resistivity of a post-annealed CuP_{2-y} film deposited on a Si_3N_4 membrane. An activation energy of (121 ± 3) meV can be extracted for the mechanism responsible for the conductivity increase above 230 K.

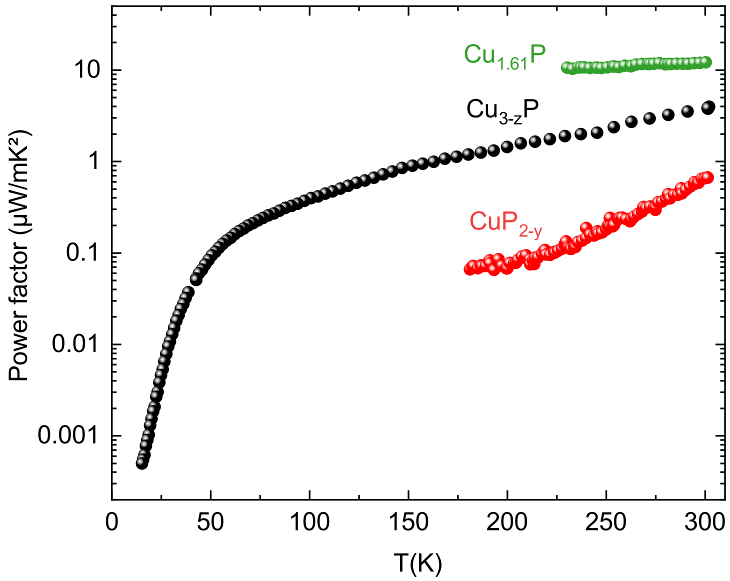

Figure S8: Temperature-dependent power factor σS^2 of the three films used for thermoelectric characterization. σ is the electrical conductivity and S is the Seebeck coefficient.

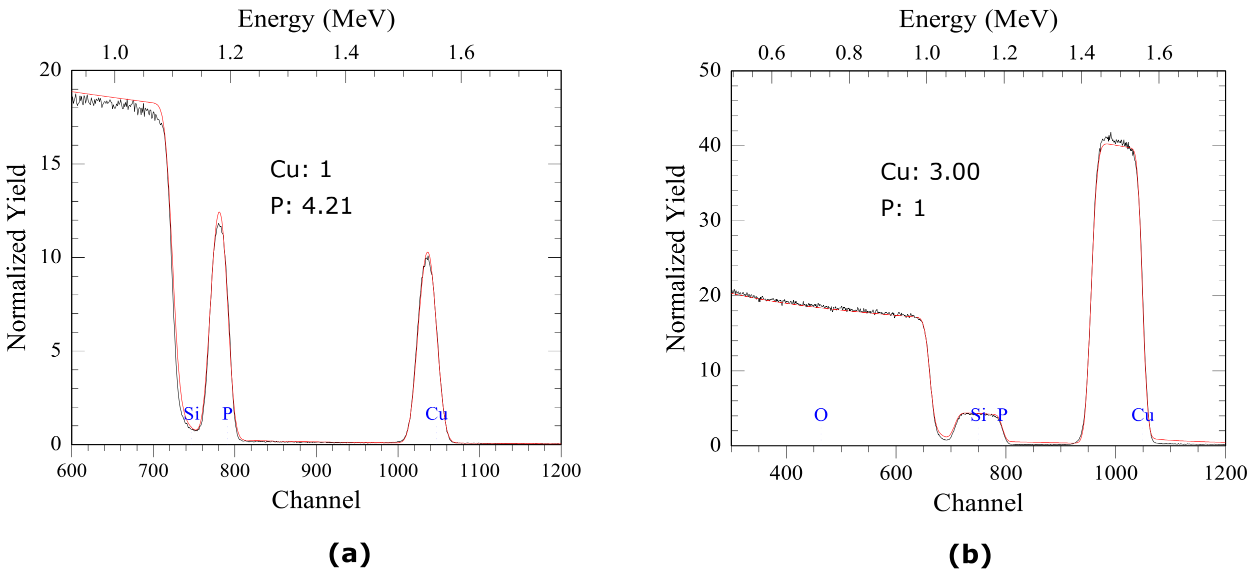

Figure S9: Examples of RBS spectra of Cu-P films used to calibrate composition measurements by XRF. For each sample, we show the fitted stoichiometric coefficients of Cu and P.

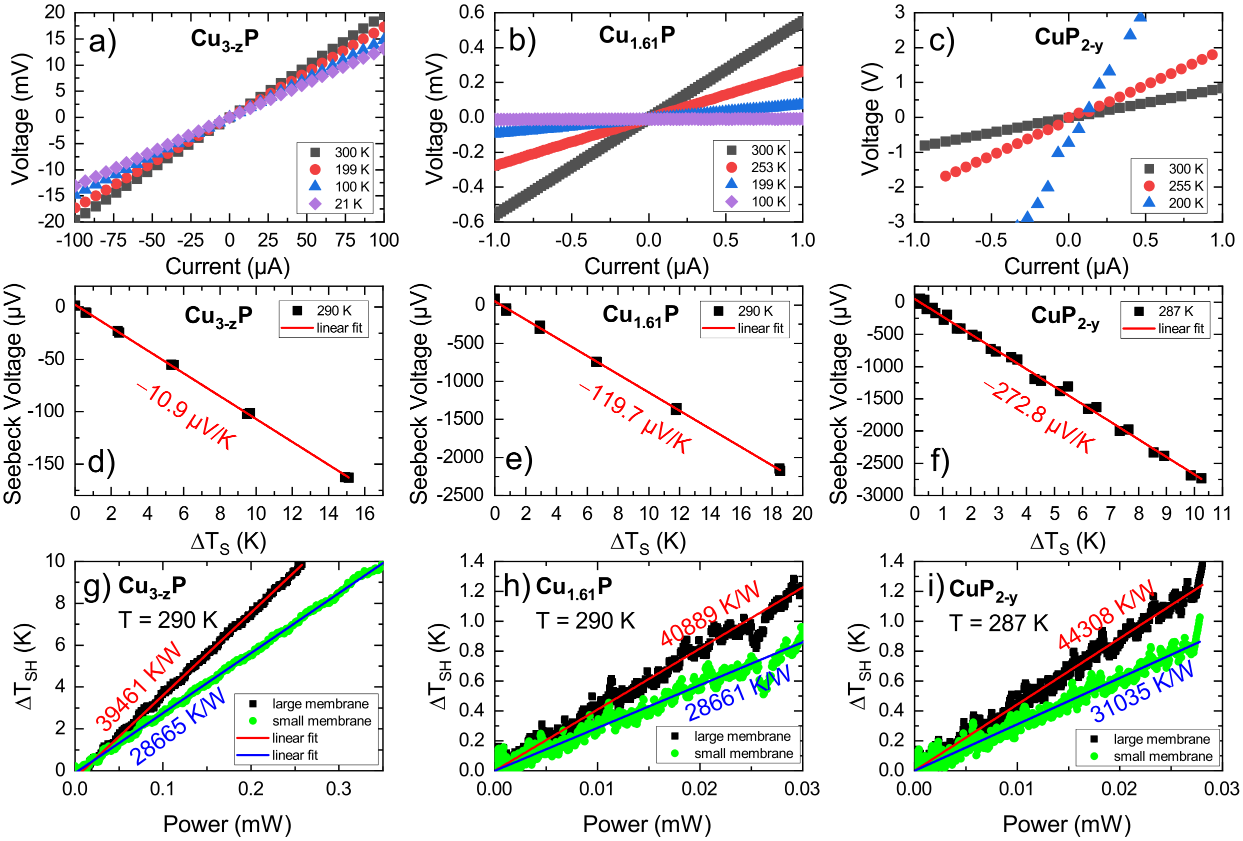

Figure S10: Processed raw data for thermoelectric characterization is shown for $Cu_{3-z}P$, $Cu_{1.61}P$ and CuP_{2-y}. (a-c) Linear behavior of I-V curves at different temperatures are shown. (d-f) The measured Seebeck voltage as function of the temperature difference is shown along with the extracted slope characterizing the relative Seebeck coefficient of the films with respect to platinum, with opposite sign. (g-i) Temperature increase of the membrane line heaters is shown as function of the applied power. Thermal resistances shown in red and blue result from the linear regression.

References

(1) Weber, A.; Sutter, P.; von Känel, H. Optical, electrical, and photoelectrical properties of sputtered thin amorphous Zn_3P_2 films. *Journal of Applied Physics* **1994**, *75*, 7448–7455.

(2) Barradas, N.; Arstila, K.; Battistig, G.; Bianconi, M.; Dytlewski, N.; Jeynes, C.; Kótai, E.; Lulli, G.; Mayer, M.; Rauhala, E.; Szilágyi, E.; Thompson, M. Summary of "IAEA intercomparison of IBA software". *Nuclear Instruments and Methods in Physics Research Section B: Beam Interactions with Materials and Atoms* **2008**, *266*, 1338–1342.

(3) Crovetto, A.; Cazzaniga, A.; Ettlinger, R. B.; Schou, J.; Hansen, O. Large process-dependent variations in band alignment and interface band gaps of Cu_2ZnSnS_4/CdS solar cells. *Solar Energy Materials and Solar Cells* **2018**, *187*, 233–240.

(4) Yi, F.; LaVan, D. A. Nanocalorimetry: Exploring materials faster and smaller. *Applied Physics Reviews* **2019**, *6*, 031302.

(5) Linseis, V.; Völklein, F.; Reith, H.; Nielsch, K.; Woias, P. Advanced platform for the in-plane ZT measurement of thin films. *Review of Scientific Instruments* **2018**, *89*, 015110.

(6) van der Pauw, L. J. A method of measuring specific resistivity and Hall effect of discs of arbitrary shape. *Philips Research Reports* **1958**, *13*, 1–9.

(7) Kockert, M.; Mitdank, R.; Zykov, A.; Kowarik, S.; Fischer, S. F. Absolute Seebeck coefficient of thin platinum films. *Journal of Applied Physics* **2019**, *126*, 105106.

(8) Völklein, F.; Reith, H.; Meier, A. Measuring methods for the investigation of in-plane and cross-plane thermal conductivity of thin films. *physica status solidi (a)* **2013**, *210*, 106–118.

(9) Kasai, A.; Abdulla, A.; Watanabe, T.; Takenaga, M. Highly Sensitive Precise Double AC Hall Effect Apparatus for Wide Resistance Range. *Japanese Journal of Applied Physics* **1994**, *33*, 4137–4145.

(10) Olofsson, O. The Crystal Structures of CuP_2 and AgP_2 with some Phase Analytical Data of the Cu-P and Ag-P Systems. *Acta Chemica Scandinavica* **1965**, *19*, 229–241.

(11) Olofsson, O. The crystal structure of Cu_3P. *Acta Chemica Scandinava* **1972**, *26*, 2777–2787.

(12) Madsen, G. K.; Carrete, J.; Verstraete, M. J. BoltzTraP2, a program for interpolating band structures and calculating semi-classical transport coefficients. *Computer Physics Communications* **2018**, *231*, 140–145.

(13) Jain, A.; Ong, S. P.; Hautier, G.; Chen, W.; Richards, W. D.; Dacek, S.; Cholia, S.; Gunter, D.; Skinner, D.; Ceder, G.; Persson, K. A. Commentary: The Materials Project: A materials genome approach to accelerating materials innovation. *APL Materials* **2013**, *1*, 011002.
